# Supplementary material for: A moisture function of soil heterotrophic respiration that incorporates microscale processes
Source: Nat Commun. 2018 Jul 2;9:2562. doi: 10.1038/s41467-018-04971-6 (PMC6028431; doi:10.1038/s41467-018-04971-6)
Supplement: Supplementary file 1 — Supplementary Information [file 41467_2018_4971_MOESM1_ESM.docx]

**A Moisture Function of Soil Heterotrophic Respiration that Incorporates Microscale Processes**

Yan et al.

**Supplementary Information**


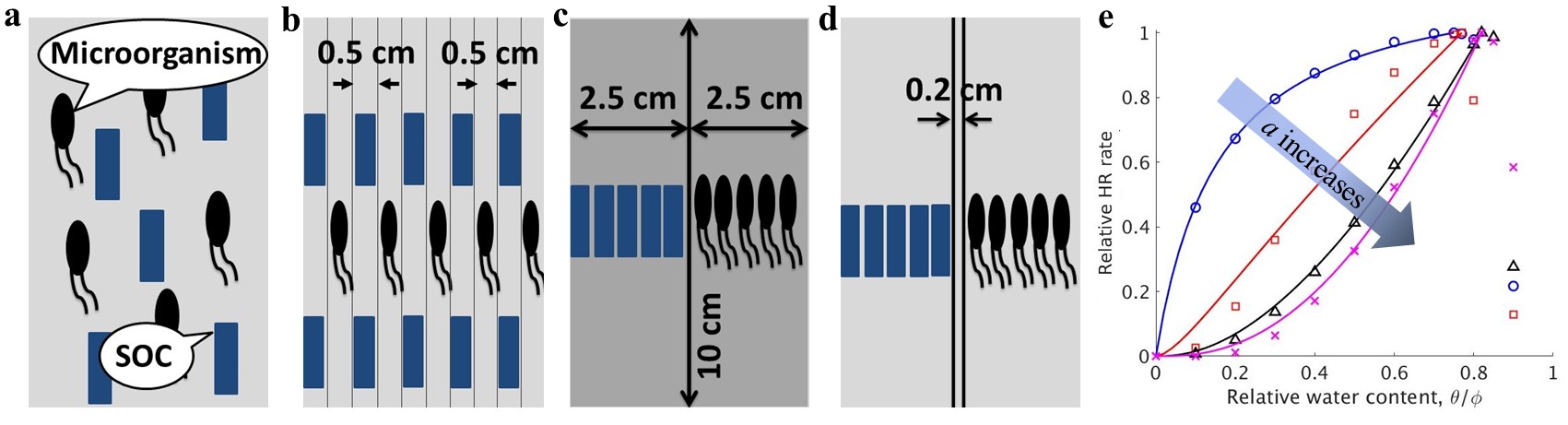


**Supplementary Figure 1. Effects of the collocation between soil-adsorbed organic carbon (SOC) and microorganisms on the** **SOC-microorganism collocation factor,** $\boldsymbol{a}$**, in the simulated soil cores with different porosity values and organic carbon contents**. (**a**-**d**), Different distributions of SOC and microorganisms in the homogenous soil cores with different porosity values $\phi$ ($\phi$ = 0.58 in **a**, **b**, **d** and 0.42 in **c**) and SOC contents *C_SOC_* (*C_SOC_* = 0.02 g g^-1^ in **a**, **c**, **d** and 0.04 g g^-1^ in **b**). (**e**) The relative heterotrophic respiration (HR) rates change with the relative water content for the different allocations of SOC and microorganisms in (**a)**-(**d**). The blue circles (○), red squares (□), black triangles (△), and pink crosses (×) represent the simulated HR rates obtained by the microscale model for the different collocations in (**a)**-(**d**). The solid curves represent the moisture function, *f_m_*, with fitted *a*, *a* =0, 0.38, 0.76 and 0.98, via the linear least square regression.


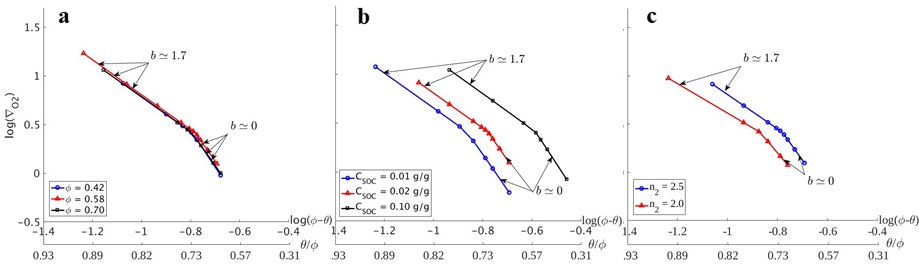


**Supplementary Figure 2.** **Effects of soil properties on the** **O_2_ supply restriction factor *b*. (a-c)** Change of $b$ with water content for the simulated homogenous soil cores with different (**a**) porosity values $\phi$, (**b**) soil-adsorbed organic carbon (SOC) contents $C_{SOC}$, and (**c**) saturation exponents $n_{g}$. The value of *b* was calculated using $b=n_{g}+1+\omega$ , where parameter *ω* is the slope of log(∇_O2_)-log(*ϕ-θ*) curves (see Eq. 8 in Methods section). The gradient of O_2_ at the soil-atmosphere interface, ∇_O2_, was calculated using the atmospheric O_2_ concentration and the simulated O_2_ concentration in the top numerical voxels of the soil cores. $\phi$ = 0.58, $C_{SOC}$ = 0.02 g g^-1^, and $n_{g}$ = 2.5 were used in the simulations, unless they were changed as indicated by the legends.


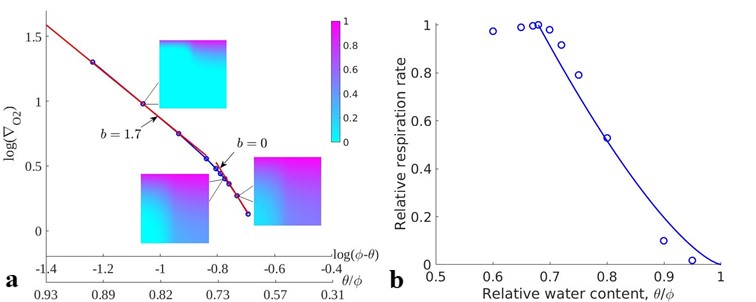


**Supplementary Figure 3. Effects of O_2_ distribution on the O_2_ supply restriction factor, *b*, in a simulated heterogenous soil core.** (**a**) Change of $b$ with water content, $b=\omega+3.5$ where parameter *ω* is the slope of log(∇_O2_)-log(*ϕ-θ*) curves (see Eq. 8 in Methods section). The gradient of O_2_ at the soil-atmosphere interface, ∇_O2_, was calculated using the atmospheric O_2_ concentration and the simulated O_2_ concentration in the top numerical voxels of the homogenous soil cores. The inset plots show the distributions of the relative O_2_ concentration with respect to the atmospheric O_2_ concentration under different relative water contents ($\theta/\phi$ = 0.85, 0.71, and 0.68). The porosity, $\phi$, is 0.42 in the left half and 0.76 in the right half of the soil core. (**b**) Comparisons between the fitted moisture function, *f_m_*, and the simulated heterotrophic respiration (HR)-moisture relationship. The blue circles (○) represent the simulation results obtained by the microscale model, and the solid line represents *f_m_* with $b$ = 1.4, which was fitted via the linear least square regression.


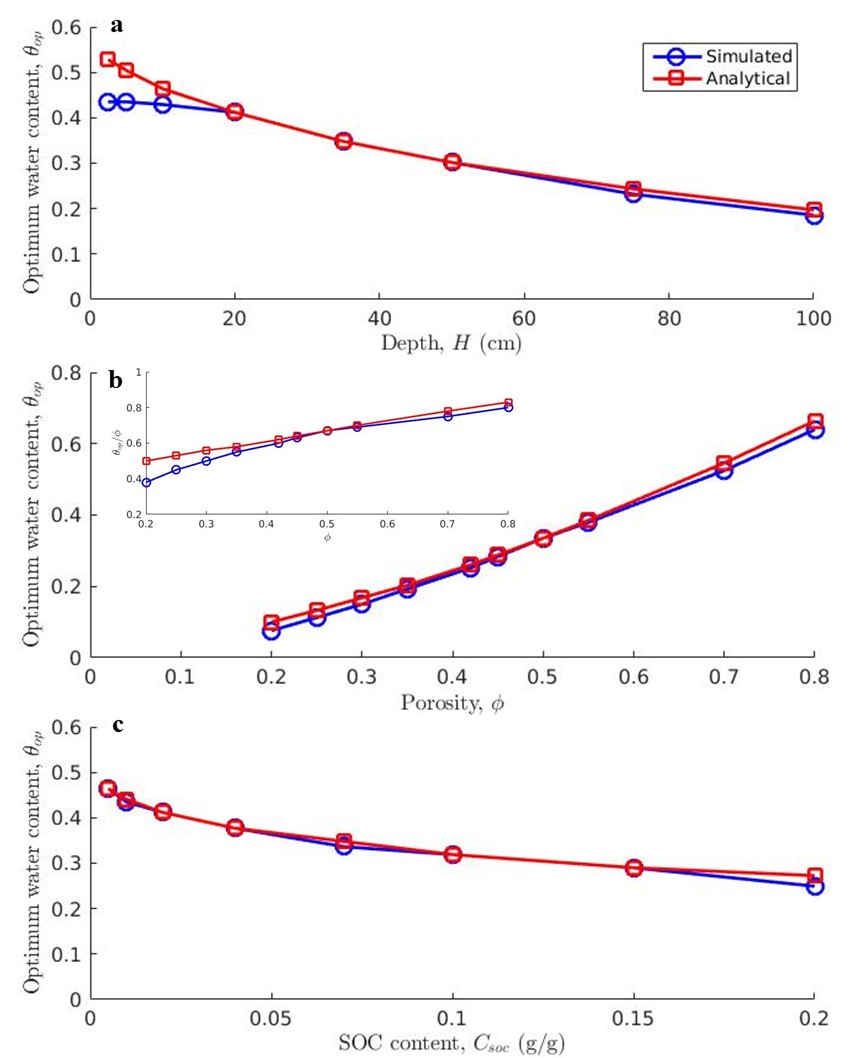


**Supplementary Figure 4. Comparisons between values of the analytical and the simulated optimum water content,** $\boldsymbol{\theta}_{\boldsymbol{op}}$**, for the simulated homogenous soil cores with different properties:** (**a**) depths, (**b**) porosity values, and (**c**) soil-adsorbed organic carbon (SOC) contents. The subplot in (**b**) represents the comparisons of the relative water content, $\theta_{op}/\phi$, for different porosity values. The values of the simulated $\theta_{op}$ were derived from the simulated heterotrophic respiration (HR)-moisture relationship obtained by the microscale model; the values of the analytical $\theta_{op}$ were calculated using Eq. (11) in Methods section. The base values were $H=20$ cm, $\phi=0.58$, and $C_{SOC}$ = 0.02 g g^-1^ in the simulations.


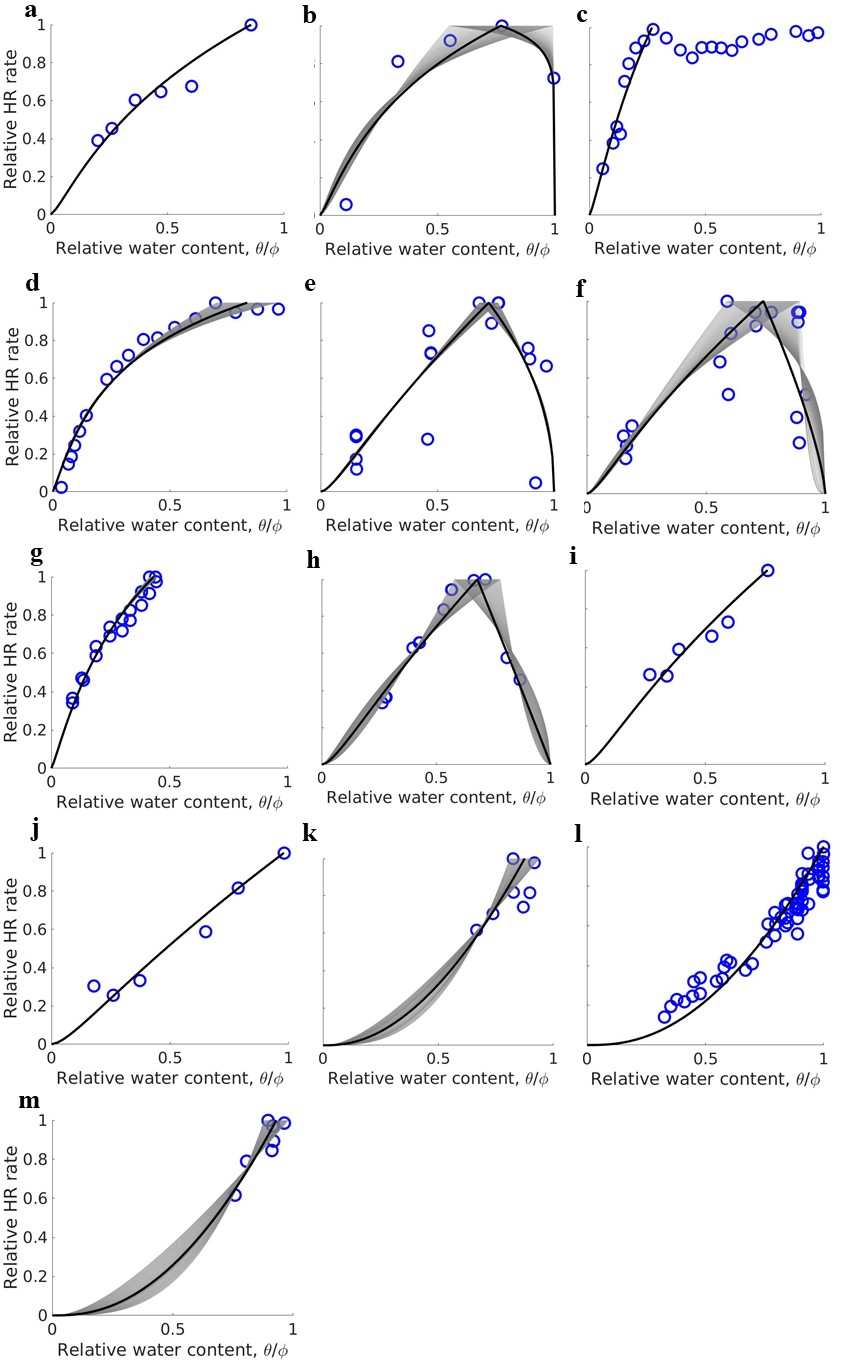


**Supplementary Figure 5. Calibration of the moisture function, *f_m_*, using a wide range of soil types.** Blue circles (○) are experimental data for different soil types: (**a**) Sand (Gleyic Cambisol, undisturbed) [^1^](#_ENREF_1), (**b**) Sand [^2^](#_ENREF_2), (**c**) Sandy loam (mineral soil) [^3^](#_ENREF_3), (**d**) Sandy loam (forest floor litter soil) [^3^](#_ENREF_3), (**e**) Sandy loam (mineral soil) [^4^](#_ENREF_4), (**f**) Sandy loam (Forest soil) [^4^](#_ENREF_4), (**g**) Sandy loam [^5^](#_ENREF_5), (**h**) Silt loam [^6^](#_ENREF_6), (**i**) Silt loam (Selhausen, undisturbed) [^1^](#_ENREF_1), (**j**) Silt loam (Merzenhausen, undisturbed) [^1^](#_ENREF_1), (**k**) Sandy clay loam [^7^](#_ENREF_7), (**l**) Clay loam [^8^](#_ENREF_8), (**m**) Clay [^7^](#_ENREF_7). Gray ranges represent the fitted *f_m_* with respect to different optimum water contents, $\theta_{op}$, whose values were allowed to vary in a range. The black lines represent the best-fitted *f_m_* using the averaged values of *a*, *b*, and $\theta_{op}$ corresponding to the gray ranges (see Supplementary Data 1).


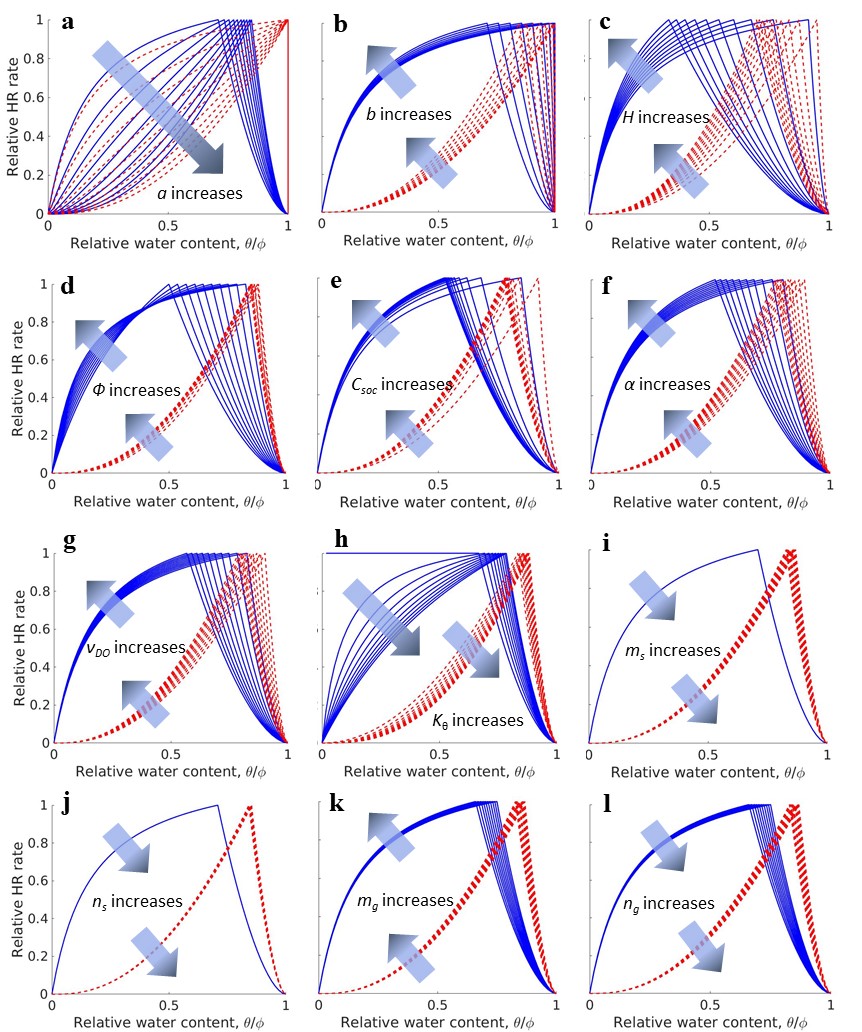


**Supplementary Figure 6. Sensitivities of the moisture function, *f_m_*, to soil properties**: (**a**) soil-adsorbed organic carbon (SOC)-microorganism collocation factor *a* (0 $\leq$ *a* $\leq$ 1, corresponding to 0.016 $\leq$ *c_c_* $\leq$ 0.37 g g^-1^ where *c_c_* is clay content); (**b**) O_2_ supply restriction factor *b* (0 $\leq$ *b* $\leq$ 1.7); (**c**) depth *H* (2.5 $\leq$ *H* $\leq$ 100 cm); (**d**) porosity $\phi$ ($0.2\leq\phi\leq0.8$); (**e)** SOC content $C_{SOC}$ ($0.005\leq C_{SOC}\leq0.2$ g g^-1^); (**f**) mass transfer coefficient $\alpha$ ($1\times{10}^{-8}\leq\alpha\leq5\times{10}^{-8}$ s^-1^); (**g**) stoichiometric coefficient $\upsilon_{DO}$ ($1\leq\upsilon_{DO}\leq5$ g g^-1^); (**h**) moisture constant $K_{\theta}$ ($0\leq K_{\theta}\leq0.5$ m^3^ m^-3^); (**i**) cementation exponent for dissolved organic carbon (DOC) diffusion $m_{s}$ ($1\leq m_{s}\leq2$); (**j**) saturation exponent for DOC diffusion $n_{s}$ ($1.5\leq n_{s}\leq2$.5); (**k**) cementation exponent for O_2_ diffusion $m_{g}$ ($1\leq m_{g}\leq2$); (**l**) saturation exponent for O_2_ diffusion $n_{g}$ ($2\leq n_{g}\leq3$). $a=0$ and 1 in the blue solid and red dash curves, respectively, in all plots except in (**a**), in which *a* changes from 0 to 1 with $b=1.7$ in the blue solid curves and $b=0$ in the red dash curves. $b=1.7$ in all plots except in (**a**) and (**b**). The base values are *H* = 20 cm, $\phi$ = 0.58, $C_{SOC}$ = 0.02 g g^-1^, $\alpha=2\times{10}^{-8}$ s^-1^, $\upsilon_{DO}$ = 2.45 g g^-1^, $K_{\theta}$ = 0.1 m^3^ m^-3^, $m_{s}$ = 1.5, $n_{s}$ = 2, $m_{g}$ = 1.5, and $n_{g}$ = 2.5.


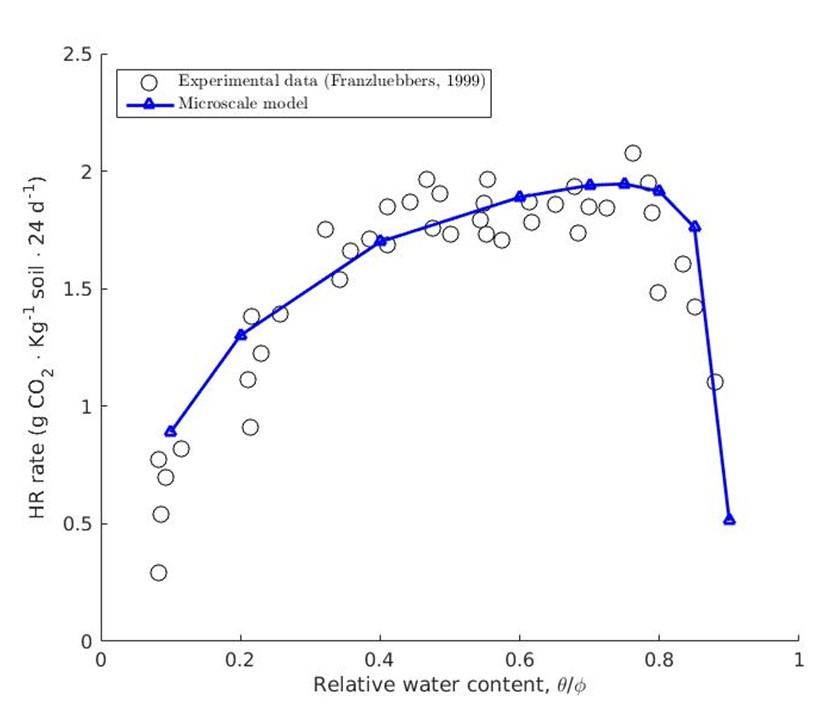


**Supplementary Figure 7. Calibration of the microscale model using experimental data**. The heterotrophic respiration (HR) rates were estimated by measuring the total CO_2_ produced in the canning jars in the experiments [^9^](#_ENREF_9); the HR rates were calculated by averaging the CO_2_ flux at the top surface of the simulated soil core in the simulations.

| Symbols | Descriptions | Values | Units | Sources and notes |
| --- | --- | --- | --- | --- |
| $\boldsymbol{\rho}_{\boldsymbol{s}}$ | Density of soil mineral | 2.65×10^3^ | kg m^-3^ | [^9^](#_ENREF_9) |
| $\boldsymbol{K}_{\boldsymbol{c}}$ | Adsorption/desorption equilibrium constant | 0.039 | l g^-1^ | [^10^](#_ENREF_10) |
| $\boldsymbol{K}_{\boldsymbol{\theta}}$ | Moisture constant | 0.1 | m^3^ m^-3^ | [^11^](#_ENREF_11) |
| $\boldsymbol{\alpha}$ | Mass transfer coefficient | 2×10^-8^ | s^-1^ | [^12^](#_ENREF_12) |
| $\boldsymbol{D}_{\boldsymbol{DOC,0}}$ | Diffusion coefficient of DOC | 1.9×10^-10^ | m^2^ s^-1^ | [^13^](#_ENREF_13) |
| $\boldsymbol{D}_{\boldsymbol{DO,0}}$ | Diffusion coefficient of DO | 2.1×10^-9^ | m^2^ s^-1^ | [^14^](#_ENREF_14) |
| $\boldsymbol{D}_{\boldsymbol{GO,0}}$ | Diffusion coefficient of gaseous O_2_ | 2.1×10^-5^ | m^2^ s^-1^ | [^15^](#_ENREF_15) |
| $\boldsymbol{D}_{\boldsymbol{DIC,0}}$ | Diffusion coefficient of DIC | 1.92×10^-9^ | m^2^ s^-1^ | [^14^](#_ENREF_14) |
| $\boldsymbol{D}_{\boldsymbol{GIC,0}}$ | Diffusion coefficient of gaseous CO_2_ | 1.92×10^-5^ | m^2^ s^-1^ | [^15^](#_ENREF_15) |
| $\boldsymbol{m}_{\boldsymbol{s}}$ | Cementation exponent for solute diffusion | 1.5 | - | [^16^](#_ENREF_16) |
| $\boldsymbol{n}_{\boldsymbol{s}}$ | Saturation exponent for solute diffusion | 2 | - | [^16^](#_ENREF_16) |
| $\boldsymbol{m}_{\boldsymbol{g}}$ | Cementation exponent for gas diffusion | 1.5 | - | [^16^](#_ENREF_16) |
| $\boldsymbol{n}_{\boldsymbol{g}}$ | Saturation exponent for gas diffusion | 2.5 | - | [^16^](#_ENREF_16) |
| $\mathbf{Y}$ | Yield coefficient | 0 | g g^-1^ | Assumed |
| $\mathbf{k}_{\mathbf{b}}$ | Decay rate | 0 | s^-1^ | Assumed |
| $\boldsymbol{k}_{\boldsymbol{DOC}}$ | Maximum reaction rate of DOC | 1.97×10^-5^ | g g^-1^ s^-1^ | [^17^](#_ENREF_17) |
| $\boldsymbol{\nu}_{\boldsymbol{DO}}$ | Stoichiometric coefficient of DO | 2.45 | g g^-1^ | [^18^](#_ENREF_18) |
| $\boldsymbol{\nu}_{\boldsymbol{DIC}}$ | Stoichiometric coefficient of DIC | 3.43 | g g^-1^ | [^18^](#_ENREF_18) |
| $\boldsymbol{K}_{\boldsymbol{DOC}}$ | Half-saturation coefficient of DOC | 1.3×10^-4^ | g l^-1^ | [^17^](#_ENREF_17) |
| $\boldsymbol{K}_{\boldsymbol{DO}}$ | Half-saturation coefficient of DO | 1.0×10^-4^ | g l^-1^ | [^17^](#_ENREF_17) |
| $\boldsymbol{K}_{\boldsymbol{h,o}}$ | Henry constant for O_2_ | 3.181×10^-2^ | - | [^19^](#_ENREF_19) |
| $\boldsymbol{K}_{\boldsymbol{h,c}}$ | Henry constant for CO_2_ | 0.2137 | - | [^19^](#_ENREF_19) |
| $\boldsymbol{K}_{\boldsymbol{a}\boldsymbol{1}}$ | Equilibrium constant between carbonic acid and bicarbonate | 10^-6.3^ | mole m^-3^ | [^20^](#_ENREF_20) |
| $\boldsymbol{K}_{\boldsymbol{a}\boldsymbol{2}}$ | Equilibrium constant between bicarbonate and carbonate | 10^-10.25^ | mole m^-3^ | [^20^](#_ENREF_20) |
| $\boldsymbol{C}_{\boldsymbol{DO,0}}$ | Initial concentration of DO | 8.3×10^-3^ | g l^-1^ | [^21^](#_ENREF_21) |
| $\boldsymbol{C}_{\boldsymbol{GO,0}}$ | Initial concentration of gaseous O_2_ | 0.2609 | g l^-1^ | [^22^](#_ENREF_22) |
| $\boldsymbol{C}_{\boldsymbol{B,0}}$ | Initial concentration of microorganism | 4.53x10^-4^ | g g^-1^ | [^23^](#_ENREF_23) |
| $\boldsymbol{C}_{\boldsymbol{DIC,0}}$ | Initial concentration of DIC | 2.93×10^-3^ | g l^-1^ | [^21^](#_ENREF_21) |
| $\boldsymbol{C}_{\boldsymbol{GIC,0}}$ | Initial concentration of gaseous CO_2_ | 7.91×10^-4^ | g l^-1^ | [^22^](#_ENREF_22) |

**Supplementary Table 1. Parameter and initial values used in the study**

| Function | $\boldsymbol{f(}\frac{\boldsymbol{\theta}}{\boldsymbol{\phi}}\boldsymbol{)}$ = | Sources |
| --- | --- | --- |
| Candy | $4\frac{\theta}{\phi}\left( 1-\frac{\theta}{\phi} \right)$, $\frac{\theta}{\phi}\ll0.5$  1, $\frac{\theta}{\phi}>0.5$ | [^24^](#_ENREF_24) |
| Century | $\frac{1}{1+30exp(-8.5\theta/\phi)}$ | [^25^](#_ENREF_25) |
| Daycent | ($\frac{\theta/\phi-b}{a-b})^{d(\frac{b-a}{a-c})}(\frac{\theta/\phi-c}{a-c})^{d}$ | [^26^](#_ENREF_26) |
| Demeter | $0.25+0.75\frac{\theta}{\phi}$ | [^27^](#_ENREF_27) |
| Gompertz | $exp(-\exp\left( a-b\frac{\theta}{\phi} \right))$ | [^28^](#_ENREF_28) |
| Moyano | $3.11\frac{\theta}{\phi}-2.42\left( \frac{\theta}{\phi} \right)^{2}$ | [^29^](#_ENREF_29) |
| Myers | $b\frac{\theta}{\phi}+(1-b)\left( \frac{\theta}{\phi} \right)^{2}$ | [^28^](#_ENREF_28) |
| Patcis | 0, $\frac{\theta}{\phi}<0.01$  $1-exp(-11.07\frac{\theta}{\phi}+0.11)$, $\frac{\theta}{\phi}\gg0.01$ | [^24^](#_ENREF_24) |
| Rothc | 0.2+1.44$\frac{\theta}{\phi}$, $\frac{\theta}{\phi}\ll0.556$  $1$, $\frac{\theta}{\phi}>0.556$ | [^24^](#_ENREF_24) |
| Skopp | $min[\alpha(\frac{\theta}{\phi})^{f},\beta(1-\frac{\theta}{\phi})^{g}$] | [^28^](#_ENREF_28) |
| Standcarb | $1-exp(-3/(\frac{\theta}{\phi})_{min}\left( \frac{\theta}{\phi}+a \right))^{b}\exp{(-\left( \frac{\theta}{\phi\left( M_{max}+c \right)} \right)}^{d})$ | [^28^](#_ENREF_28) |

**Supplementary Table 2. Empirical moisture functions used in Earth system models (ESMs)**

**Supplementary References**

1. Herbst, M., Tappe, W., Kummer, S. & Vereecken, H. The impact of sieving on heterotrophic respiration response to water content in loamy and sandy topsoils. *Geoderma* **272**, 73-82 (2016).

2. Wickland, K. P. & Neff, J. C. Decomposition of soil organic matter from boreal black spruce forest: environmental and chemical controls. *Biogeochemistry* **87**, 29-47 (2008).

3. Lee, X., Wu, H.-J., Sigler, J., Oishi, C. & Siccama, T. Rapid and transient response of soil respiration to rain. *Global Change Biol* **10**, 1017-1026 (2004).

4. Bowden, R. D., Newkirk, K. M. & Rullo, G. M. Carbon dioxide and methane fluxes by a forest soil under laboratory-controlled moisture and temperature conditions. *Soil Biology and Biochemistry* **30**, 1591-1597 (1998).

5. Liu, W., Zhang, Z. H. E. & Wan, S. Predominant role of water in regulating soil and microbial respiration and their responses to climate change in a semiarid grassland. *Global Change Biol* **15**, 184-195 (2009).

6. Skopp, J., Jawson, M. D. & Doran, J. W. Steady-State Aerobic Microbial Activity as a Function of Soil Water Content. *Soil Science Society of America Journal* **54**, 1619-1625 (1990).

7. Schjønning, P., Thomsen, I. K., Moldrup, P. & Christensen, B. T. Linking Soil Microbial Activity to Water- and Air-Phase Contents and Diffusivities. *Soil Science Society of America Journal* **67**, 156-165 (2003).

8. Cook, F. J., Orchard, V. A. & Corderoy, D. M. Effects of lime and water content on soil respiration. *New Zealand Journal of Agricultural Research* **28**, 517-523 (1985).

9. Franzluebbers, A. J. Microbial activity in response to water-filled pore space of variably eroded southern Piedmont soils. *Applied Soil Ecology* **11**, 91-101 (1999).

10. Jardine, P. M., McCarthy, J. F. & Weber, N. L. Mechanisms of Dissolved Organic Carbon Adsorption on Soil. *Soil Science Society of America Journal* **53**, 1378-1385 (1989).

11. Yan, Z., Liu, C., Todd-Brown, K. E., Liu, Y., Bond-Lamberty, B. & Bailey, V. L. Pore-scale investigation on the response of heterotrophic respiration to moisture conditions in heterogeneous soils. *Biogeochemistry* **131**, 121-134 (2016).

12. Zarnetske, J. P., Haggerty, R., Wondzell, S. M., Bokil, V. A. & González-Pinzón, R. Coupled transport and reaction kinetics control the nitrate source-sink function of hyporheic zones. *Water Resources Research* **48**, (2012).

13. Hendry, M. J., Ranville, J. R., Boldt-Leppin, B. E. J. & Wassenaar, L. I. Geochemical and transport properties of dissolved organic carbon in a clay-rich aquitard. *Water Resources Research* **39**, WR001943 (2003).

14. Cussler, E. L. *Diffusion*. Cambridge University Press (1997).

15. Weast, R. C. *CRC Handbook of Chemistry and Physics*. CRC Press (1997).

16. Hamamoto, S., Moldrup, P., Kawamoto, K. & Komatsu, T. Excluded-volume expansion of Archie's law for gas and solute diffusivities and electrical and thermal conductivities in variably saturated porous media. *Water Resources Research* **46**, 6514 (2010).

17. Borden, R. C. & Bedient, P. B. Transport of dissolved hydrocarbons influenced by oxygen-limited biodegradation: 1. Theoretical development. *Water Resources Research* **22**, 1973-1982 (1986).

18. Yan, Z., Liu, C., Todd-Brown, K. E., Liu, Y., Bond-Lamberty, B. & Bailey, V. L. Pore-scale investigation on the response of heterotrophic respiration to moisture conditions in heterogeneous soils. *Biogeochemistry*, 1-14.

19. Sander, R. Compilation of Henry's law constants (version 4.0) for water as solvent. *Atmos Chem Phys* **15**, 4399-4981 (2015).

20. Stumm, W. & Morgan, J. J. *Aquatic chemistry: chemical equilibria and rates in natural waters*. Wiley (1996).

21. Colt, J. Front-matter. In: *Computation of Dissolved Gas Concentration in Water as Functions of Temperature, Salinity and Pressure (Second Edition)*. Elsevier (2012).

22. Wallace, J. M. & Hobbs, P. V. *Atmospheric Science: An Introductory Survey*. Academic Press (1977).

23. Powlson, D. & Jenkinson, D. A comparison of the organic matter, biomass, adenosine triphosphate and mineralizable nitrogen contents of ploughed and direct-drilled soils. *The Journal of Agricultural Science* **97**, 713-721 (1981).

24. Bauer, J., Herbst, M., Huisman, J. A., Weihermüller, L. & Vereecken, H. Sensitivity of simulated soil heterotrophic respiration to temperature and moisture reduction functions. *Geoderma* **145**, 17-27 (2008).

25. Parton, W., Morgan, J., Kelly, R. & Ojima, D. Modeling soil C responses to environmental change in grassland systems. *The potential of US grazing lands to sequester carbon and mitigate the greenhouse effect Lewis Publ, New York*, 371-398 (2000).

26. Kelly, R. H., Parton, W. J., Hartman, M. D., Stretch, L. K., Ojima, D. S. & Schimel, D. S. Intra-annual and interannual variability of ecosystem processes in shortgrass steppe. *Journal of Geophysical Research: Atmospheres* **105**, 20093-20100 (2000).

27. Foley, J. A. An equilibrium model of the terrestrial carbon budget. *Tellus B* **47**, 310-319 (1995).

28. Sierra, C. A., Trumbore, S. E., Davidson, E. A., Vicca, S. & Janssens, I. Sensitivity of decomposition rates of soil organic matter with respect to simultaneous changes in temperature and moisture. *Journal of Advances in Modeling Earth Systems* **7**, 335-356 (2015).

29. Moyano, F. E., Manzoni, S. & Chenu, C. Responses of soil heterotrophic respiration to moisture availability: An exploration of processes and models. *Soil Biology and Biochemistry* **59**, 72-85 (2013).
