# Supplementary material for: A moisture function of soil heterotrophic respiration that incorporates microscale processes
Source: Nat Commun. 2018 Jul 2;9:2562. doi: 10.1038/s41467-018-04971-6 (PMC6028431; doi:10.1038/s41467-018-04971-6)
Supplement: Supplementary file 3 — Description of Additional Supplementary Files [file 41467_2018_4971_MOESM3_ESM.pdf]

Description of Additional Supplementary Files

File Name: Supplementary Data 1
Description: The dataset 1 contains basic soil properties and estimated parameter values related to the moisture function f_m for a wide range of soil types.

File Name: Supplementary Software 1
Description: The Software 1 is used to calculate the optimum water content θ_{op}.

File Name: Supplementary Software 2
Description: Software 2 is used to determine and plot the moisture function f_p.
