# Supplementary material for: A moisture function of soil heterotrophic respiration that incorporates microscale processes
Source: Nat Commun. 2018 Jul 2;9:2562. doi: 10.1038/s41467-018-04971-6 (PMC6028431; doi:10.1038/s41467-018-04971-6)
Supplement: Supplementary file 5 — Supplementary Software 1 [file 41467_2018_4971_MOESM5_ESM.docx]

Matlab Code #1: calculate optimum water content $\theta_{op}$

function calculate_theta_op

% calculate the optimum water content (theta_op) using equation (10) in Methods

%% parameter initialization (parameter values change with soil characteristics)

phi = 0.58;

c = 0.0; % g/g

m1 = 1.5;

n1 = 2;

m2 = 1.5;

n2 = 2.5;

nu_DO = 2.45; % g/g

K_theta = 0.1; % m^3/m^3

alpha = 2e-8; % 1/s

rho_s = 2.65e3; % kg/m^3

H = 0.2; % m

C_soc = 0.02; % g/g

D_GO = 2.1e-5; % m^2/s

%% calculate SOC-microorganism collocation factor (a) according to clay content (c)

% for heterogenous soils

% if c<0.036

% a = 0;

% elseif c > 0.34

% a = 1;

% else

% a = 3.31*c - 0.12;

% end

% for homogeneous soils

a = 0;

%% parameter b is assumed constant

% b = 1.7 for homogeneous soils

% b = 0.75 for heterogenous soils

b = 1.7;

%% calculate total mass of SOC per unit surface area (m_soc)

% m_soc can also be given directly

m_soc = rho_s*(1-phi)*H*C_soc;

%% calculate optimal water content (theta_op)

function f=symfun(x)

f = nu_DO*x/(K_theta+x)*alpha*m_soc*phi^(a*(m1-n1))*x^(a*n1)-(1.72*C_soc+0.065)*phi^(m2-n2)*(phi-x)^b*D_GO;

end

theta_op = bisection(@symfun,0,phi,1e-9)

end

%% sub-function

function p = bisection(f,a,b,eps)

if f(a)*f(b)>0

disp('Wrong choice bro')

else

p = (a + b)/2;

err = abs(f(p));

while err > eps

if f(a)*f(p)<0

b = p;

else

a = p;

end

p = (a + b)/2;

err = abs(f(p));

end

end
