# Supplementary material for: A moisture function of soil heterotrophic respiration that incorporates microscale processes
Source: Nat Commun. 2018 Jul 2;9:2562. doi: 10.1038/s41467-018-04971-6 (PMC6028431; doi:10.1038/s41467-018-04971-6)
Supplement: Supplementary file 6 — Supplementary Software 2 [file 41467_2018_4971_MOESM6_ESM.docx]

Matlab Code #2: determine moisture function *f_p_*

function function_fp

%% plot moisture function (fp)

% the x-axis was changed to relative water content (theta_op/phi)

phi = 0.58; % value change with soil characteristics

c = 0.0; % can be changed, unit: g/g

n1 = 2;

K_theta = 0.1; % unit: m^3/m^3

%% calculate SOC-microorganism collocation factor (a) according to clay content (c)

% for heterogenous soils

% if c<0.036

% a = 0;

% elseif c > 0.34

% a = 1;

% else

% a = 3.31*c - 0.12;

% end

% for homogeneous soils

a = 0;

%% parameter b is assumed constant

% b = 1.7 for homogeneous soils

% b = 0.75 for heterogenous soils

b = 1.7;

%% theta_op is calculated by the function calculate_theta_op in Supplementary Code 1

s_op = theta_op/phi

n1 = 40;

n2 = 20;

a_th = 0.0;

a_ep = 0.;

n = 2.5;

sl = linspace(0,s_op,n1);

sr = linspace(s_op,1,n2);

s12 = [sl sr];

th1 = phi*sl;

th2 = phi*sr;

th_op = phi*s_op;

for i = 1:n1

fth(i) = (K_theta+th_op)/th_op^(1+a*n1)*th1(i)^(1+a*n1)/(K_theta+th1(i));

end

for i = 1:n2

fth(n1+i) = (phi-th2(i))^b/(phi-th_op)^b;

end

figure,hold on

set(gcf,'Units','centimeters','Position',[0 0 20 16])

set(gca,'FontSize',20)

plot(s12,fth,'b-','LineWidth',2)

ylabel('Relative respiration rate') % label x-axis

xlabel('Relative water content, \theta/\phi') % label right y-axis

axis([0 1 0 1.])
